# Association of Circulating microRNAs in Plasma with Coronary Collateral Circulation in Patients with Chronic Total Occlusion

**DOI:** 10.3390/ijms27209116

**Published:** 2026-10-14

**Authors:** Anna Kiseleva, Evgeniia Sotnikova, Anastasia Zharikova, Dmitry Vasilev, Vladimir Kutsenko, Anna Borisova, Alexandra Ershova, Alexey Meshkov, Oxana Drapkina

**Affiliations:** 1 National Medical Research Center for Therapy and Preventive Medicine, Ministry of Healthcare of the Russian Federation, Petroverigsky Per. 10, Bld. 3, Moscow 101000, Russia; sotnikova.evgeniya@gmail.com (E.S.);; 2 Faculty of Bioengineering and Bioinformatics, Lomonosov Moscow State University, 1-73, Leninskie Gory, Moscow 119991, Russia

**Keywords:** miRNA, chronic total occlusion, coronary collateral circulation, coronary artery disease, plasma

## Abstract

Chronic total occlusion (CTO) occurs in 16–52% of patients with coronary artery disease (CAD) and is often accompanied by variable development of coronary collateral circulation (CCC). The aim of this work was to study the associations between circulating plasma microRNAs (miRNAs) and the development of CCC in patients with CTO. This exploratory study included 43 patients: a control group—patients without significant coronary artery stenosis (*n* = 20), and two CTO groups with good (*n* = 13) and poor (*n* = 10) CCC based on the Rentrop classification. The study was performed simultaneously on two types of plasma samples (K2EDTA and sodium citrate). Total RNA was isolated using the miRNeasy Serum/Plasma Advanced Kit. Libraries were prepared using the QIAseq miRNA UDI Library Kit and then sequenced on the NextSeq 550 using a 1 × 75 bp protocol. A total of 18 miRNAs were found to be differentially expressed (DE) in EDTA plasma and 13 in citrate plasma; only one miRNA in citrate plasma (hsa-miR-92a-3p) was differentially expressed between the good and poor CCC groups. However, given the limited sample size, the findings should be considered hypothesis-generating, and further studies are required for validation in larger, independent cohorts and to establish the roles of these miRNAs in CCC development.

## 1. Introduction

Chronic total occlusion of a coronary artery (CTO) is defined as the absence of antegrade blood flow with an estimated occlusion duration of at least 3 months [1]. CTO is a condition identified in 16–52% of patients with coronary artery disease (CAD) [2,3,4]. Unlike patients with non-occlusive forms of CAD, individuals with CTO more often have severe comorbid conditions: diabetes mellitus, arterial hypertension, peripheral vascular disease, and a history of myocardial infarction [2]. In response to CTO, coronary collateral circulation (CCC) develops as a natural bypass pathway that maintains myocardial blood supply [5]. Well-developed CCC is associated with preserved cardiac function, higher survival rate, and a lower risk of adverse cardiac events in patients with CTO and CAD [6,7,8,9]. The extent of CCC is commonly graded using the Rentrop classification during coronary angiography [10].

The assessment of CCC development is primarily invasive (e.g., during coronary angiography), which limits repeated evaluation and large-scale screening. Therefore, identifying non-invasive biomarkers that can distinguish patients with good versus poor CCC is an important clinical goal [11]. Circulating microRNAs (miRNAs) are promising candidates in this context. miRNAs are small, non-coding, highly conserved single-stranded RNAs approximately 22 nucleotides in length that regulate gene expression and are stable in human biofluids [12], making standardized biosamples from biobank collections an excellent source for miRNA studies. They play important roles in complex diseases, including CAD, and are involved in the regulation of angiogenesis, arteriogenesis, and vascular remodeling [6,11,13,14]. Several miRNAs have been associated with CCC development or CTO in previous studies [6,7,8,11,13,14,15], most of which investigated only a small number of miRNAs using quantitative real-time polymerase chain reaction (qPCR). However, data on the association of circulating miRNAs with different degrees of CCC development remain limited and heterogeneous [15]. Importantly, previous studies differ substantially in sample type (whole blood, plasma, or serum), anticoagulant used, and storage duration. These preanalytical and analytical factors can strongly influence circulating miRNA profiles and complicate cross-study comparisons [16]. In particular, EDTA and citrate plasma may differ in miRNA yield, library quality, and the number of detected miRNAs, especially after long-term storage. A systematic comparison of plasma anticoagulants and storage conditions is therefore needed to improve reproducibility in miRNA studies [17,18,19].

The aim of this exploratory study was to investigate the associations between circulating miRNAs and the development of CCC in patients with CTO using two types of plasma samples.

## 2. Results

### 2.1. Comparison of Plasma miRNA Expression Levels Between Patient Groups

The study included 43 patients, who were divided into three groups: patients with CAD and CTO of a large epicardial coronary artery (diameter > 2.5 mm), subdivided into groups with good (*n* = 13) and poor (*n* = 10) development of CCC using the Rentrop classification [10,20], and a control group that included patients without significant coronary artery stenosis (*n* = 20). Clinical and demographic characteristics of patients are presented in Table 1 and Supplementary Table S1. Given the small subgroup sizes, the statistical power of these comparisons is limited, and the findings should therefore be interpreted with caution.

Next-generation sequencing (NGS) characteristics of the study groups are presented in Table 2. When comparing the CTO group and the control group for both EDTA and citrate plasma samples, statistically significant differences were obtained for plasma and NGS library storage times. For detected miRNAs per sample, statistically significant differences were detected only for EDTA plasma samples in three comparison groups; when comparing the poor CCC group and the control group, there were no statistically significant differences detected. Plasma storage time was the longest for the good CCC group that is consistent with the smallest number of detected miRNAs. The difference in plasma storage time was almost perfectly confounded with CCC status, as good CCC and poor CCC samples were accrued in two temporally distinct batches. Consequently, storage time could not be adjusted for as a covariate in the differential expression analysis, and some of the observed miRNA differences may reflect preanalytical storage-time effects rather than CCC-related biology.

Principal component analysis (PCA) based on human miRNA gene expression data (according to miRBase [21]) revealed relative homogeneity across the study groups (Supplementary Figure S1).

**Figure 1:**
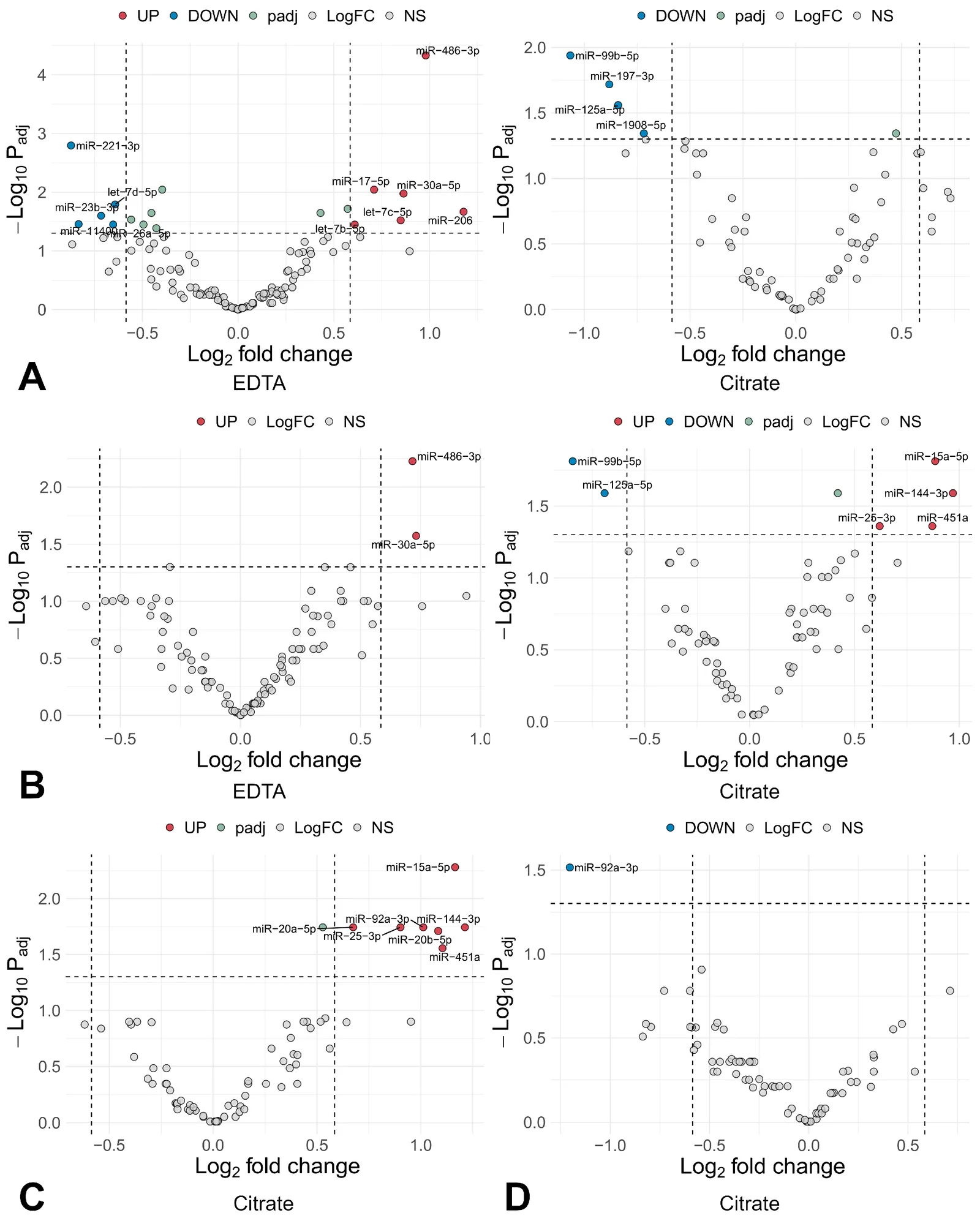
DE miRNAs in patient comparison groups. (**A**) Good CCC vs. control group; (**B**) CTO vs. control group; (**C**) poor CCC vs. control group; (**D**) good CCC vs. poor CCC. For all miRNAs in the graph, gray indicates miRNAs for which an insignificant change in expression was detected (*p*adj > 0.05); blue—*p*adj < 0.05 and a greater-than-1.5-fold decrease in expression; red—*p*adj < 0.05 and a greater-than-1.5-fold increase in expression; green—*p*adj < 0.05 and a less-than-1.25-fold change in expression; CCC—coronary collateral circulation; CTO—chronic total occlusion of a coronary artery; *p*adj—*p*-values adjusted for multiple comparisons.

Using NGS data, differential expression of human miRNAs was analyzed (Table 3, Figure 1). There were no differentially expressed (DE) miRNAs in EDTA plasma samples when comparing the good and poor CCC groups, as well as the poor CCC and control groups.

A total of 18 DE miRNAs in EDTA plasma and 13 DE miRNAs in citrate plasma were identified between the comparison groups (Table 4 and Supplementary Tables S2–S7). Of the 31 DE miRNAs, ten were identified in more than one comparison group: two for EDTA plasma samples (hsa-miR-30a-5p, hsa-miR-486-3p); eight for citrate plasma samples (hsa-miR-15a-5p, hsa-miR-25-3p, hsa-miR-92a-3p, hsa-miR-99b-5p, hsa-miR-125a-5p, hsa-miR-144-3p, hsa-miR-451a, hsa-let-7i-5p). The miRNA hsa-miR-92a-3p had different expression directions: it was downregulated in the good CCC group in comparison with the poor CCC group, and it was upregulated in the poor CCC group when compared with controls. No overlapping DE miRNAs were found between samples derived from different plasma types. Of the 18 DE EDTA plasma miRNAs, 11 had a greater-than-1.5-fold change in expression; of the 13 DE citrate plasma miRNAs, 11 also had a greater-than-1.5-fold change in expression. No correction for multiple comparisons was applied across the different group comparisons.

For all DE miRNAs that showed associations with CCC development or CTO, functional analysis was performed [23,24,25]. According to the TargetScanHuman database [25], there were no common targets for 18 DE miRNAs in EDTA plasma and 13 DE miRNAs in citrate plasma. Enrichment analysis of the predicted mRNA targets using the Gene Ontology (GO) database [23] and the Kyoto Encyclopedia of Genes and Genomes (KEGG) pathway database [24] did not reveal any significantly enriched biological processes or pathways.

### 2.2. Comparison of Plasma miRNA Expression Levels Between Different Anticoagulants

Analysis using Bioanalyzer 2100 (Agilent, CA, USA) revealed that the average height of the peak corresponding to adapter dimers was relatively high (approximately 50% of the height of the miRNA peak). For citrate plasma samples, the adapter dimer peak was higher than for EDTA plasma samples. Later it was established that the high adapter dimer percentage could be at least partially caused by the use of QIAseq miRNA 96 Index Kit IL UDI-A due to the technical, transportation, and/or storage issues [26]. Unfortunately, additional purification from dimers using polyacrylamide gel electrophoresis could not be performed. Notably, handling long-stored citrate plasma samples during NGS library preparation was considerably more difficult. Therefore, to obtain libraries of sufficient quality, library preparation had to be prepared several times. No such problems were observed with EDTA samples.

**Figure 2:**
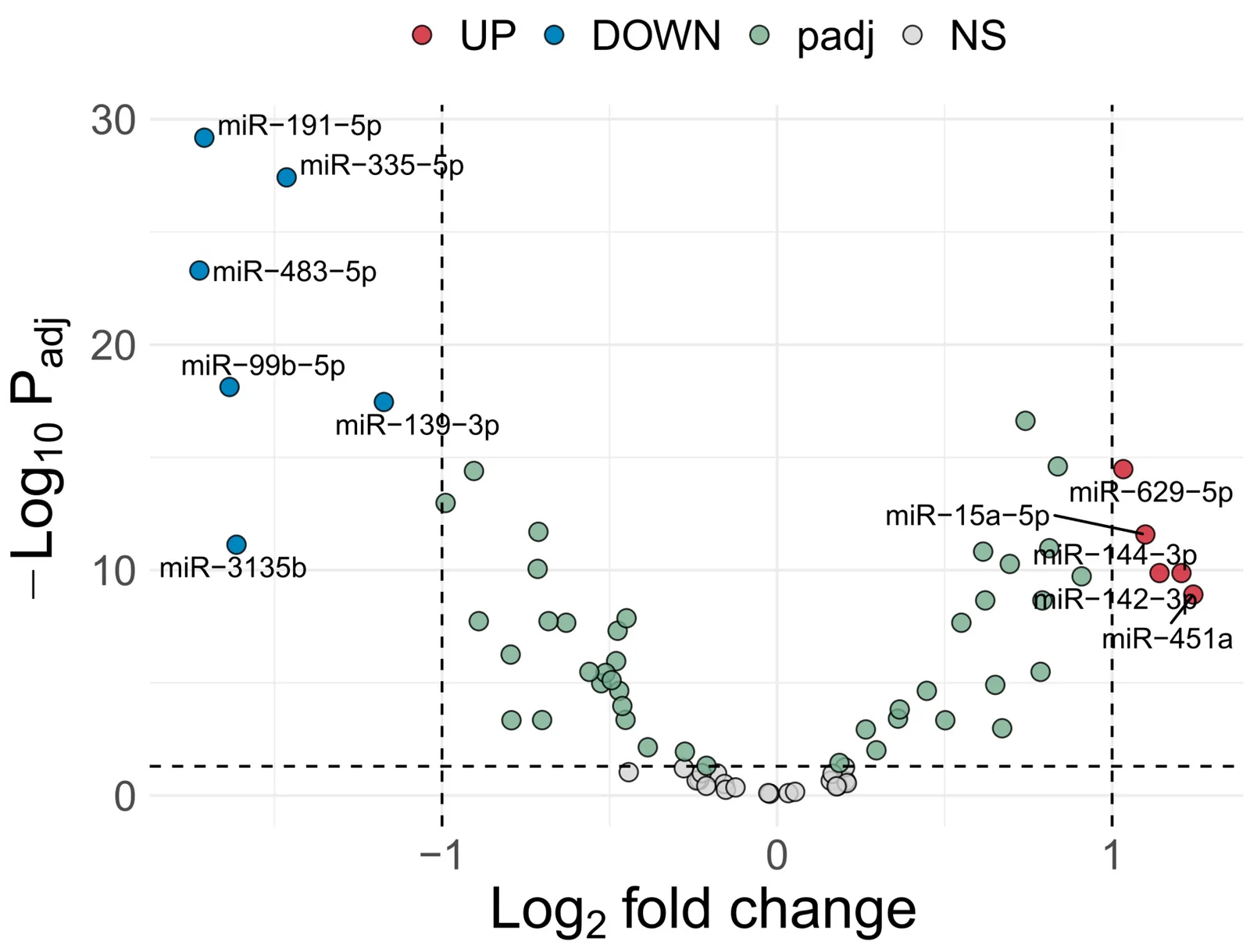
DE miRNAs in EDTA and citrate samples. For all miRNAs in the graph, gray indicates miRNAs for which an insignificant change in expression was detected (*p*adj > 0.05); blue—*p*adj < 0.05 and a greater-than-2-fold decrease in expression; red—*p*adj < 0.05 and a greater-than-1.5-fold increase in expression; green—*p*adj < 0.05 and a less-than-1.25-fold change in expression; *p*adj—*p*-values adjusted for multiple comparisons.

NGS analysis results are presented in Table 5. The PCA data showed the absence of homogeneity across EDTA and citrate plasma samples (Supplementary Figure S2). Differential expression of miRNAs between samples with various anticoagulants is presented in Figure 2. A total of 53 DE miRNAs were detected between EDTA and citrate samples (Supplementary Table S8).

## 3. Discussion

To provide an objective prioritization, we applied a threshold of absolute fold change > 1.5. Under these criteria, of the 18 DE EDTA plasma miRNAs, 11 had a greater-than-1.5-fold change in expression; and 11 of the 13 DE citrate plasma miRNAs had the same. However, we used this threshold only for prioritization, not to dismiss other DE miRNAs. No independent validation was performed in this study, either in the same cohort using a different method or in an independent cohort. Therefore, the identified DE miRNAs should be regarded as exploratory and hypothesis-generating.

The limited sample size (*n* = 43, with only 13 and 10 patients in the good and poor CCC groups, respectively) is another limitation of this study. The study was not powered to detect small-to-moderate effect sizes, and the likelihood of false-negative findings is substantial. However, the median sample size of earlier published CTO and CCC studies is about 60 participants [15], with the largest of 330 [11] and the smallest of 26 [7]. Only three of them used NGS for analysis [7,8,14] with sample sizes of 9–26 participants; others used qPCR [6,11,13,15].

Of all DE miRNAs found in the present study, only seven have been previously described in studies on CTO patients (hsa-miR-23b-3p, hsa-miR-26a-5p, hsa-miR-126-3p, hsa-let-7d-5p, hsa-miR-15b-5p, hsa-miR-92a-3p, hsa-miR-451a) [9,14,22]. All three studies have substantial design differences that include comparison groups and sample type (EDTA plasma [22], plasma (anticoagulant unspecified) [14], and whole blood [9]). Moreover, in the study by Vural et al. (2024), the transcoronary gradient (the difference between whole blood miRNA expression in the aortic root and the right atrium or coronary sinus) [9] was assessed, which makes it impossible to compare their results to the results of other circulating miRNA studies that used plasma or serum samples obtained with a less invasive procedure.

The only DE miRNA common to the study by Zhu et al. [14] and the present study was hsa-miR-15b-5p. However, in the study by Zhu et al. [14], hsa-miR-15b-5p was downregulated in plasma (anticoagulant unspecified) in the good CCC group compared to the poor CCC group and to the healthy controls, as well as in the poor CCC group compared to the healthy controls. In the present study, hsa-miR-15b-5p was upregulated in the poor CCC group compared with the controls (patients without significant coronary artery stenosis), but there was no statistically significant difference between the good CCC and poor CCC groups.

In the present study, in citrate plasma samples, hsa-miR-92a-3p, which, as shown earlier, is involved in angiogenesis [27], was downregulated in the good CCC group compared to the poor CCC group, while upregulated in the poor CCC group compared to the controls. In contrast, a previous study [13] reported no statistically significant differences in hsa-miR-92a-3p levels between the good and poor CCC groups.

In our previous study, EDTA plasma samples from all 43 participants were analyzed for eight miRNAs (hsa-miR-126-5p, hsa-miR-146a-5p, hsa-miR-155-5p, hsa-miR-15b-5p, hsa-miR-21-5p, hsa-miR-23a-3p, hsa-miR-451a, hsa-miR-503-5p) using TaqMan assays. Statistically significant differences in expression were observed for five miRNAs (hsa-miR-126-5p, hsa-miR-155-5p, hsa-miR-15b-5p, hsa-miR-23a-3p, hsa-miR-451a) between the good CCC group and the controls, and for two miRNAs (hsa-miR-126-5p, hsa-miR-23a-3p) between the CTO group and the controls [22].

In the present NGS-based study, however, only two of these miRNAs (hsa-miR-15b-5p, hsa-miR-451a) showed statistically significant differential expression. The miRNA hsa-miR-15b-5p was downregulated in EDTA plasma in the good CCC group compared to the control group under TaqMan analysis [22], while NGS analysis showed its upregulation in citrate plasma in the poor CCC group compared to the control group. For miR-451a, TaqMan analysis showed increased expression in EDTA plasma when comparing the good CCC group with the control group [22]. In contrast, NGS data revealed increased expression levels only in citrate plasma when comparing the CTO group with the control group, as well as the poor CCC group with the controls. It is worth mentioning that hsa-miR-451a is frequently used for hemolysis evaluation [28]. In this study, the absence of hemolysis was assessed visually for all the samples; additionally, hemolysis was not detected in any EDTA samples via qPCR analysis of hsa-miR-23a-3p and hsa-miR-451a [22].

The present study had a median EDTA plasma storage time before RNA extraction of 38.0 [35.0; 60.0] months, followed by RNA storage for a median of 21 [20; 21] days before analysis. In contrast, in a TaqMan-based study, the median storage time of EDTA plasma before RNA extraction was 2 [1; 20] months, and the median storage time of RNA before analysis—1 [1; 3] day [22]. Consequently, the observed differences in estimated expression levels of the eight miRNAs between these two studies may be attributed to the substantially longer storage periods for both plasma and extracted RNA in the current study.

It was shown that a wide range of preanalytical and analytical factors affect the analysis of circulating miRNA [16,29]. One of them is the type of sample (serum or plasma); another is the type of anticoagulant used for plasma collection. Although sodium citrate and EDTA remain the most widely used anticoagulants in studies of circulating miRNA (based on the studies included in our previous reviews [15,30]), reports on their effects on miRNA levels remain inconsistent. For instance, Mussbacher et al. (2020) observed an increase in miRNA levels over time in EDTA plasma samples at room temperature [17], and Zhelankin et al. (2022) found that the use of EDTA blood collection tubes induced higher hemolysis levels in plasma samples compared to other anticoagulants [18]. On the contrary, Suzuki et al. (2022) reported higher erythrocyte miRNA levels in citrate and fluoride samples and consequently preferred EDTA for the analysis [19].

The number of reads mapped to genes and the number of detected miRNAs per sample were higher in EDTA plasma than in citrate plasma samples. Differential expression between EDTA and citrate plasma was shown for 53 miRNAs. Additionally, the quality of miRNA libraries (e.g., low adapter dimer percentage) was also higher for EDTA plasma samples compared to citrate plasma. This suggests that the use of citrate as an anticoagulant for long-term plasma storage may affect the circulating miRNAs more substantially than the use of EDTA does.

This lack of concordance in disease-associated miRNAs between EDTA and citrate plasma types, together with the significant differences in storage-related and NGS-related parameters between these sample types, underscores a critical limitation in biomarker development: the circulating miRNA profile is highly dependent on the preanalytical factors, potentially reflecting anticoagulant-specific biochemical effects, and findings derived from one specific plasma type cannot be assumed to be directly transferable to another without cross-validation [17,18,19].

Another important factor is storage time. The difference in plasma storage time between the good CCC group and both the poor CCC group and the control group is a major confounder that cannot be statistically adjusted for, because storage time is almost perfectly collinear with CCC status. Therefore, the observed associations between specific miRNAs and CCC should be interpreted with caution, since we cannot exclude the possibility that some of these associations are driven by preanalytical rather than biological factors. Literature on the impact of long-term plasma storage at −70 °C on circulating miRNA levels is sparse and contradictory [19,29,31,32]. Preanalytical variables related to sample storage time likely contributed to the low replication rate. In designing the present study, we proceeded on the assumption that circulating miRNAs are more stable during long-term storage in whole plasma than in extracted aqueous solutions, based on our previous findings [33]. However, despite this presumed stability, the significant time gap between the two experimental phases and the prolonged storage duration of the plasma samples still could profoundly affect the current results. Furthermore, the majority of published studies fail to explicitly report the exact duration of sample storage. It is rather difficult to assess the reproducibility of studies that use biosamples with varying or undocumented storage times. To ensure the reliability and cross-study comparability of circulating miRNA biomarkers, future investigations must account for, standardize, and transparently report these preanalytical variables, including precise storage durations.

Notably, most of the aforementioned miRNA association studies either used short-term stored plasma or did not mention the storage duration. To our knowledge, there are no comparative data on the impact of different anticoagulants on circulating miRNAs during long-term storage. In this study, plasma samples were stored for about 3 years before NGS analysis.

The commercial kits used for NGS library preparation can also contribute to differences in the DE miRNAs found. The QIAseq miRNA UDI Library Kit we chose is widely used in miRNA studies and showed good performance in comparison studies [34,35]. All these preanalytical factors make cross-study comparison considerably more difficult and underscore the need to systematically account for storage time of plasma and miRNA samples to improve the reproducibility of miRNA studies [16,33].

This study has several limitations, in particular a limited sample size, absence of validation with independent methods in either the same or a new cohort and on short-stored biosamples, and relatively small library size per sample. The samples were retrieved from an existing institutional biobank and had been collected during routine clinical care; storage duration and batch assignment reflected the original clinical sampling and biobank workflow rather than a prospective study design. In particular, good CCC and poor CCC samples were accrued in two temporally distinct batches, which was not under the control of the investigators. Because plasma storage time differed markedly between CCC groups and was almost perfectly confounded with CCC status, we could not adjust for storage time as a covariate; therefore, we cannot exclude the possibility that some observed miRNA differences reflect preanalytical storage-time effects rather than CCC-related biology. Angina and lipid-lowering therapy could not be adjusted for in the CTO vs. control comparison due to complete separation; these factors therefore remain potential residual confounders.

Furthermore, functional enrichment analysis of the DE miRNAs most strongly associated with CTO and CCC development did not identify any cardiovascular-specific GO terms or KEGG pathways, suggesting these miRNAs might reflect general CTO, general ischaemic or inflammatory processes, or preanalytical and technical variability rather than CCC extent specifically. The purpose of TargetScan filtering was to identify precisely those DE microRNAs that could subsequently be considered as potential biomarkers; moreover, miRNAs can act through non-canonical targets and cell-type-specific mechanisms. Thus, the reported associations should be considered exploratory and hypothesis-generating and should not be interpreted as validated biomarkers. Independent validation in larger cohorts, short-stored biosamples, using standardized preanalytical protocols, and functional experiments will be required before any clinical application can be considered.

## 4. Materials and Methods

### 4.1. Study Subjects

The study included 43 patients examined at the National Medical Research Center (NMRC) for Therapy and Preventive Medicine (Moscow, Russia) [22], who were divided into three groups. The first, a control group, consisted of patients without significant coronary artery stenosis (*n* = 20). Patients with CTO of a large epicardial coronary artery (diameter > 2.5 mm) (CTO group), subdivided into second and third groups: those with good CCC (*n* = 13, good CCC group) and those with poor CCC (*n* = 10, poor CCC group) based on the Rentrop classification [10,20]. All study participants underwent coronary angiography (angiography system, General Electric, Waukesha, WI, USA). The samples were retrieved from an existing institutional biobank and had been collected during routine clinical care; storage duration and batch assignment reflected the original clinical sampling and biobank workflow rather than a prospective study design. In particular, good CCC and poor CCC samples were accrued in two temporally distinct batches, which was not under the control of the investigators.

Inclusion criteria were: (1) for the control group—age over 18 years, suspected CAD, and absence of significant coronary artery stenosis; (2) for the CTO group with well-developed CCC—age over 18 years, presence of angina symptoms, known CTO and planned percutaneous coronary intervention, and presence of CTO of a large epicardial artery (diameter > 2.5 mm) with good CCC, noted as Rentrop grade 3, based on the degree of collateral filling of the occluded vessel; and (3) for the CTO group with poor CCC—age over 18 years, presence of angina symptoms, known CTO and planned percutaneous coronary intervention, and CTO of a large epicardial artery (diameter > 2.5 mm) with poor CCC noted as Rentrop grade 0.

Exclusion criteria were: acute coronary syndrome (ACS) within the last 6 months; chronic infection; history of oncological disease; inflammatory diseases; psychiatric disorders; pregnancy; and refusal to sign informed consent.

The study design and previous studies involving the same cohort are presented in Table 6.

### 4.2. Ethical Statement

The study was done according to the Declaration of Helsinki and the National Standard of the Russian Federation “Good Clinical Practice (GCP)” (GOST R 52379-2005) [36] and was approved by the Independent Ethics Committee of the NMRC for Therapy and Preventive Medicine (protocols number 05-05/15, dated 9 June 2015, and number 02-02/22, dated 17 March 2022). To comply with these regulations, as well as Article 93 of the Federal Law “On the Fundamentals of Health Protection of Citizens of the Russian Federation” (dated 21 November 2011, No. 323-FZ), written informed consent for the processing of their personal data was obtained from all participants. To ensure participant confidentiality, all clinical, biochemical, and genetic data were de-identified and encrypted.

### 4.3. Blood and Plasma Collection

Tubes containing K2EDTA or sodium citrate (3.2%) were used for blood collection. Collected blood was stored at room temperature for no more than one hour prior to plasma separation. Then, blood was centrifuged for 15 min at 1200× *g* at +4 °C (5702 R, Eppendorf, Hamburg, Germany), after which plasma was aliquoted and stored at –70 °C (DF 590, Nuve, Ankara, Turkey) without freeze–thaw cycles. The detection of hemolysis was done visually for all plasma samples. In addition, hemolysis was not detected in any EDTA samples via qPCR analysis of hsa-miR-23a-3p and hsa-miR-451a, as previously described [22].

### 4.4. RNA Isolation

Plasma samples were thawed on ice prior to RNA extraction and centrifuged at 14,000× *g* for 15 min at +4 °C. The supernatant was then transferred to clean, nuclease-free tubes and immediately used for RNA isolation using the miRNeasy Serum/Plasma Advanced Kit (Qiagen, Hilden, Germany). When isolating RNA from EDTA plasma, the protocol for EDTA plasma was used. RNA from citrate plasma was isolated using two protocols—for citrate plasma and for EDTA plasma. Since there were no significant differences in concentration, RNA samples obtained using the EDTA plasma protocol were used for library preparation and subsequent sequencing in order to ensure uniformity [16]. QIAseq miRNA Library QC Spike-Ins (Qiagen, Germany) were mixed with RPL buffer before being added to the plasma samples at a concentration of 0.5 µL spike-in per sample. Immediately after isolation, each obtained RNA sample was aliquoted and stored at –70 °C until further analysis to avoid freeze–thaw cycles. The concentration of isolated miRNA was determined using a Qubit 4 fluorometer (Thermo Fisher Scientific, Waltham, MA, USA) with the Qubit microRNA Assay Kit (Thermo Fisher Scientific, Waltham, MA, USA).

### 4.5. Next-Generation Sequencing

Libraries for sequencing were prepared using the QIAseq miRNA UDI Library Kit (Qiagen, Germany). Dilution of primers and 3′ and 5′ adapters was performed according to the “Serum/Plasma” version of the manufacturer’s protocol using the QIAseq miRNA 96 Index Kit IL UDI-A. The concentration of the prepared libraries was determined using a Qubit 4 fluorometer (Thermo Fisher Scientific, MA, USA) with the Qubit 1X dsDNA HS Assay Kit. Library length was estimated using Bioanalyzer 2100 (Agilent, Santa Clara, CA, USA). Sequencing was carried out on the NextSeq 550 platform (Illumina, San Diego, CA, USA) according to the 1 × 75 bp protocol. For samples below the lower quartile by library size, resequencing of the initial NGS libraries was done.

### 4.6. Bioinformatic Analysis

The main stages of bioinformatic analysis of the NGS results of the small RNAs were described earlier [37]. Bioinformatic analysis was performed using R version 4.2. For UMI processing, the UMI-tools package version 1.1.6 [38] was used. Using cutadapt version 5.2 [39], reads of at least 14 nucleotides in length were retained. Single-end reads in FASTQ format were aligned to miRBase [21] using STAR (2.7.10a_alpha_220506) with the parameter alignEndsType EndToEnd [40]. The alignment files were sorted and indexed using Samtools version 1.2.1 [41]. Gene annotation was performed using featureCounts (v2.0.1) [42] using the following parameters: –primary. The miRBase (http://www.mirbase.org/) gene annotation was used [21]. For visualization of the results, the R package ggplot2 version 4.0.3 [43] was used. After quality control (small library size), one citrate plasma sample from the good CCC group was excluded from further analysis.

Only miRNAs that had more than 50 reads in at least 10 samples (for patient groups) and 40 samples (for anticoagulant groups) were taken for the differential expression analysis. This threshold was chosen to reduce noise from low-abundance miRNAs while retaining sufficient miRNAs for subsequent analysis. Differences between groups in expression levels of miRNAs were assessed using the standard tests from the DESeq2 package (version 1.42.1) [44]. For the revealed DE miRNAs, functional analysis was performed [23,24,25]. The predicted targets for DE miRNAs were obtained using the TargetScanHuman database [25] (with filters of context score <−0.3 and context score percentile > 0.5). For the obtained messenger RNA targets, enrichment analysis was carried out based on the Gene Ontology (GO) gene annotation database [23] for the Biological Process (BP) category and the Kyoto Encyclopedia of Genes and Genomes (KEGG) pathway database [24].

### 4.7. Statistical Analysis

Statistical analysis was performed using R version 4.2. Continuous variables are presented as median and interquartile range: Me [Q1; Q3]; discrete variables are presented as absolute and relative frequencies. Continuous variables were compared using the Mann–Whitney U test, and categorical variables using Fisher’s exact test. Differences were considered statistically significant at *p* < 0.05. Adjustment for multiple comparisons was performed using the Benjamini–Hochberg procedure within the DESeq2 package (version 1.42.1) [44]. Multiple-testing correction was applied within each DESeq2 comparison. No additional correction for multiple comparisons was applied across the different group comparisons in the miRNA differential expression analysis (four subgroups and two plasma types) because the tests across plasma types and subgroups are not independent: miRNA levels are clearly correlated across plasma types, and applying a standard global correction across all tests would be overly conservative and would substantially reduce statistical power. In addition, DESeq2 does not provide a straightforward way to incorporate an arbitrary cross-comparison correlation structure into a single full-family adjustment.

## 5. Conclusions

This study demonstrates the association with CTO for 18 DE miRNAs in EDTA plasma and 13 DE miRNAs in citrate plasma samples; in EDTA plasma, 11 of the 18 DE miRNAs had a greater-than-1.5-fold change; in citrate plasma, 11 of the 13 DE miRNAs had a greater-than-1.5-fold change. Only hsa-miR-92a-3p was differentially expressed between the good and poor CCC groups. However, given the limited sample size, all DE miRNAs should be considered exploratory and hypothesis-generating. Independent validation in larger, well-powered cohorts with standardized preanalytical protocols is required to confirm the prognostic power of these miRNAs before any clinical application can be considered.

## Figures and Tables

**Table 1:** Clinical and demographic characteristics.

Parameter	CTO (*n* = 23)		Control Group (*n* = 20)	*p*-Value Good CCC vs. Poor CCC	*p*-Value Good CCC vs. Control Group	*p*-Value Poor CCC vs. Control Group	*p*-Value CTO vs. Control Group
	Good CCC (*n* = 13)	Poor CCC (*n* = 10)					
Men, n (%)	9/13 (69.2%)	10/20 (50.0%)	0.34	0.31	0.049	0.064	9/13 (69.2%)
Age, years, Me [Q25; 75]	64.0 [56.0; 74.0]	62.5 [58.8; 69.2]	0.44	0.26	0.95	0.49	64.0 [56.0; 74.0]
BMI, kg/m^2^, Me [Q25; 75]	28.5 [25.0; 30.0]	28.1 [24.2; 30.6]	0.31	0.91	0.19	0.41	28.5 [25.0; 30.0]
Smoking, n (%)	3/13 (23.1%)	5/20 (25.0%)	0.22	0.99	0.23	0.53	3/13 (23.1%)
Family history of CVD, n (%)	9/13 (69.2%)	9/20 (45.0%)	0.10	0.28	0.69	0.76	9/13 (69.2%)
Chronic heart failure, n (%)	5/13 (38.5%)	6/20 (30.0%)	0.21	0.71	0.056	0.22	5/13 (38.5%)
Angina, n (%)	13/13 (100%)	14/20 (70.0%)	0.99	0.06	0.074	0.0064 *	13/13 (100%)
Atrial fibrillation, n (%)	4/13 (30.8%)	2/20 (10.0%)	0.34	0.18	0.99	0.42	4/13 (30.8%)
Heart rhythm disturbance, n (%)	3/13 (23.1%)	10/20 (50.0%)	0.99	0.16	0.44	0.13	3/13 (23.1%)
COPD/BA, n (%)	0/13 (0%)	3/20 (15.0%)	0.43	0.26	0.99	0.32	0/13 (0%)
Arterial hypertension, n (%)	11/13 (84.6%)	17/20 (85.0%)	0.49	0.99	0.53	0.65	11/13 (84.6%)
Post-infarction cardiosclerosis, n (%)	7/13 (53.8%)	2/20 (10.0%)	0.38	0.014	0.00029 *	0.00041 *	7/13 (53.8%)
Multifocal atherosclerosis, n (%)	7/13 (53.8%)	8/20 (40.0%)	0.99	0.49	0.71	0.54	7/13 (53.8%)
Diabetes mellitus, n (%)	0/13 (0%)	2/20 (10.0%)	0.99	0.51	0.54	0.21	0/13 (0%)
Chronic kidney disease, n (%)	3/13 (23.1%)	2/20 (10.0%)	0.99	0.36	0.58	0.42	3/13 (23.1%)
Lipid-lowering therapy, n (%)	13/13 (100%)	8/13 (61.5%)	0.99	0.039	0.046	0.0034 *	13/13 (100%)
TC, mmol/L, Me [Q25; 75]	5.2 [3.8; 5.4]	4.6 [3.4; 4.9]	0.10	0.31	0.48	0.84	5.2 [3.8; 5.4]
LDL-C, mmol/L, Me [Q25; 75]	2.5 [2.2; 3.5]	2.4 [1.6; 3.0]	0.37	0.32	0.93	0.58	2.5 [2.2; 3.5]
HDL-C, mmol/L, Me [Q25; 75]	1.1 [0.9; 1.3]	1.3 [1.2; 1.4]	0.55	0.23	0.038	0.048	1.1 [0.9; 1.3]
TG, mmol/L, Me [Q25; 75]	1.1 [1.0; 1.5]	1.1 [0.9; 1.7]	0.16	0.85	0.17	0.35	1.1 [1.0; 1.5]

BMI—body mass index; CAD—coronary artery disease; CCC—coronary collateral circulation; COPD/BA—chronic obstructive pulmonary disease/bronchial asthma; CTO—chronic total occlusion of a coronary artery; CVD—cardiovascular disease; HDL-C—high-density lipoprotein cholesterol; LDL-C—low-density lipoprotein cholesterol; Me—median; TC—total cholesterol; TG—triglycerides; Q—quartile; *—*p*-value < 0.05 after adjustment for multiple comparisons.

**Table 2:** NGS characteristics of comparison groups.

Parameter	Anticoagulant	CTO (*n* = 23)		Control Group (*n* = 20)	*p*-Value Good CCC vs. Poor CCC	*p*-Value Good CCC vs. Control Group	*p*-Value Poor CCC vs. Control Group	*p*-Value CTO vs. Control Group
		Good CCC (*n* = 13)	Poor CCC (*n* = 10)					
Plasma storage time (−70 °C) before RNA extraction, months	EDTA	59.00 [59.00; 60.00]	38.00 [37.25; 38.75]	24.50 [11.00; 37.25]	0.011	0.0017 *	0.021	<0.001 *
	Citrate	60.00 [59.75; 61.00]	38.50 [38.00; 39.75]	25.00 [12.00; 38.25]	0.013	0.0023 *	0.023	0.0011 *
RNA storage time (−70 °C) before library preparation, days	EDTA	20.00 [20.00; 21.00]	21.00 [21.00; 21.00]	20.00 [20.00; 21.00]	0.03	0.53	0.044	0.52
	Citrate	1.00 [1.00; 1.50]	4.00 [3.25; 4.75]	4.00 [4.00; 5.25]	0.012	0.0013 *	0.53	0.015
NGS library storage time before sequencing, days	EDTA	15.00 [15.00; 15.00]	14.00 [14.00; 14.00]	14.00 [14.00; 14.00]	0.0013 *	<0.001 *	0.97	0.005 *
	Citrate	13.00 [13.00; 13.00]	9.00 [9.00; 12.00]	9.00 [8.50; 9.00]	0.028	0.0013 *	0.17	0.0043 *
miRNA concentration, ng/µL, Me [Q25; 75]	EDTA	0.82 [0.68; 1.11]	0.85 [0.76; 0.96]	0.80 [0.72; 0.98]	0.88	0.84	0.7	0.71
	Citrate	0.51 [0.45; 0.56]	0.73 [0.61; 1.05]	0.66 [0.54; 0.76]	0.016	0.03	0.29	0.43
NGS library concentration, ng/µL, Me [Q25; 75]	EDTA	2.82 [2.04; 3.82]	8.10 [5.74; 8.50]	6.19 [4.60; 7.83]	<0.001 *	<0.001 *	0.13	0.13
	Citrate	1.42 [1.19; 1.79]	1.88 [1.59; 3.70]	1.60 [1.37; 2.03]	0.14	0.45	0.26	0.89
Reads per sample (library size), million, Me [Q25; 75]	EDTA	0.99 [0.87; 1.08]	1.26 [1.23; 1.33]	1.21 [0.98; 1.40]	0.13	0.057	0.95	0.19
	Citrate	2.05 [1.83; 2.33]	1.12 [0.73; 1.56]	1.06 [0.88; 1.67]	0.043	0.048	0.59	0.35
Spike-in, %	EDTA	6.72 [5.58; 12.00]	6.45 [4.13; 7.77]	5.62 [4.19; 7.49]	0.34	0.094	0.71	0.19
	Citrate	11.47 [9.68; 21.93]	10.47 [6.35; 12.82]	11.96 [10.47; 13.89]	0.2	0.69	0.17	0.61
Mapped reads, million, Me [Q25; 75]	EDTA	0.32 [0.22; 0.44]	0.66 [0.54; 0.70]	0.66 [0.55; 0.70]	0.0025 *	<0.001 *	0.75	0.0057 *
	Citrate	0.12 [0.09; 0.13]	0.11 [0.07; 0.15]	0.12 [0.10; 0.15]	1	0.41	0.65	0.42
Detected miRNAs per sample (≥50 reads per miRNA), Me [Q25; 75]	EDTA	74.00 [66.00; 87.00]	103.50 [85.75; 109.25]	100.00 [93.00; 111.25]	0.0063 *	<0.001 *	0.66	0.0074 *
	Citrate	51.00 [44.00; 55.00]	58.00 [49.25; 71.75]	64.50 [52.75; 68.50]	0.31	0.011	0.83	0.076

CCC—coronary collateral circulation; CTO—chronic total occlusion of a coronary artery; Me—median; NGS—next-generation sequencing; miRNA—microRNA; Q—Quartile; UMI—unique molecular identifiers; *—*p*-value < 0.05 after adjustment for multiple comparisons.

**Table 3:** The number of DE miRNAs associated with CCC development and CTO.

Characteristic	Good CCC vs. Poor CCC		Good CCC vs. Control Group		Poor CCC vs. Control Group		CTO vs. Control Group	
Anticoagulant	EDTA	Citrate	EDTA	Citrate	EDTA	Citrate	EDTA	Citrate
Number of DE miRNAs	0	1	18	5	0	8	2	7
Number of upregulated miRNAs	0	0	8	1	0	8	2	5
Number of downregulated miRNAs	0	1	10	4	0	0	0	2

**Table 4:** The list of DE miRNAs associated with CCC development and CTO.

miRNA	Good CCC vs. Poor CCC	Good CCC vs. Control Group	Poor CCC vs. Control Group	CTO vs. Control Group	Reference
EDTA, expression direction|Fold change/*p*adj					
hsa-miR-17-5p	—	up|1.63/0.0091	—	—	
hsa-miR-23b-3p	—	down|−1.64/0.025	—	—	[9]
hsa-miR-24-3p	—	down|−1.37/0.023	—	—	
hsa-miR-26a-5p	—	down|−1.57/0.036	—	—	[9]
hsa-miR-30a-5p	—	up|1.82/0.011	—	up|1.66/0.027	
hsa-miR-30e-3p	—	down|−1.47/0.029	—	—	
hsa-miR-126-3p	—	down|−1.34/0.041	—	—	[9,22]
hsa-miR-151a-3p	—	down|−1.41/0.036	—	—	
hsa-miR-181b-5p	—	up|1.35/0.023	—	—	
hsa-miR-206	—	up|2.26/0.022	—	—	
hsa-miR-221-3p	—	down|−1.83/0.0016	—	—	
hsa-miR-421	—	down|−1.32/0.0091	—	—	
hsa-miR-425-5p	—	up|1.49/0.019	—	—	
hsa-miR-486-3p	—	up|1.97/0.019	—	up|1.64/0.0059	
hsa-miR-11400	—	down|−1.78/0.035	—	—	
hsa-let-7b-5p	—	up|1.52/0.036	—	—	
hsa-let-7c-5p	—	up|1.80/0.030	—	—	
hsa-let-7d-5p	—	down|−1.56/0.016	—	—	[9]
Citrate, expression direction|Fold change/*p*adj					
hsa-miR-15a-5p	—	—	up|2.24/0.0052	up|1.85/0.015	—
hsa-miR-15b-5p	—	—	up|1.44/0.018	—	[14,22]
hsa-miR-20a-5p	—	—	up|1.60/0.018	—	—
hsa-miR-20b-5p	—	—	up|2.21/0.019	—	—
hsa-miR-25-3p	—	—	up|1.87/0.018	up|1.54/0.044	—
hsa-miR-92a-3p	down|−2.3/0.031	—	up|2.02/0.018	—	[9,14]
hsa-miR-99b-5p	—	down|−2.1/0.012	—	down|−1.79/0.015	—
hsa-miR-125a-5p	—	down|−1.79/0.028	—	down|−1.62/0.026	—
hsa-miR-144-3p	—	—	up|2.32/0.018	up|1.96/0.026	—
hsa-miR-197-3p	—	down|−1.84/0.019	—	—	—
hsa-miR-451a	—	—	up|2.15/0.028	up|1.83/0.044	[22]
hsa-miR-1908-5p	—	down|−1.65/0.045	—	—	—
hsa-let-7i-5p	—	up|1.39/0.045	—	up|1.34/0.026	—

CCC—coronary collateral circulation; CTO—chronic total occlusion of a coronary artery.

**Table 5:** NGS characteristics of plasma samples with different anticoagulants.

Characteristic	Citrate (*n* = 42)	EDTA (*n* = 43)	*p*-Value
Plasma storage time (−70 °C) before RNA extraction, months	39.00 [36.00; 60.00]	38.00 [35.00; 60.00]	0.50
RNA storage time (−70 °C) before library preparation, days	4.00 [1.00; 4.00]	21.00 [20.00; 21.00]	<0.001
NGS library storage time before sequencing, days	9.00 [9.00; 13.00]	14.00 [14.00; 15.00]	<0.001
miRNA concentration, ng/µL	0.60 [0.52; 0.78]	0.82 [0.72; 1.00]	<0.001
NGS library concentration, ng/µL	1.60 [1.26; 2.04]	5.00 [3.58; 7.91]	<0.001
Reads per sample (library size), million, Me [Q25; 75]	1.32 [0.88; 2.18]	1.20 [0.95; 1.34]	0.11
Spike-in, %, Me [Q25; 75]	11.43 [9.49; 13.78]	5.98 [4.33; 8.38]	<0.001
Mapped reads, million, Me [Q25; 75]	0.12 [0.10; 0.14]	0.58 [0.39; 0.69]	<0.001
Detected miRNAs per sample (≥50 reads per miRNA), Me [Q25; 75]	55.50 [49.25; 67.75]	95.00 [81.50; 107.50]	<0.001

Me—median; NGS—next-generation sequencing; miRNA—microRNA; Q—Quartile.

**Table 6:** Study design.

Study Description	Study Participants (*n* = 43)					
	Control Group (*n* = 20)		CTO Group (*n* = 23)			
			Good CCC Group (*n* = 13)		Poor CCC Group (*n* = 10)	
Anticoagulant	EDTA (*n* = 20)	Citrate (*n* = 20)	EDTA (*n* = 13)	Citrate (*n* = 13)	EDTA (*n* = 10)	Citrate (*n* = 10)
TaqMan qPCR study [22], *n*	20	-	13	-	10	-
NGS storage study [33], *n*	2	-	8	-	-	-
This NGS study, *n*	20	20	13	13	10	10

CCC—coronary collateral circulation; CTO—chronic total occlusion of a coronary artery; NGS—next-generation sequencing; qPCR—quantitative real-time polymerase chain reaction.

## Data Availability

The data used in this study, including individual information, are not publicly available due to the rules of the Ethics Committee of the National Medical Research Center for Therapy and Preventive Medicine. De-identified data can be made available upon reasonable request directed to the corresponding author, Anna Kiseleva (akiseleva@gnicpm.ru), or the Ethics Committee of the National Medical Research Center for Therapy and Preventive Medicine (phone number +7-499-5536810, secretarynec@gnicpm.ru). Data access proposals will be reviewed and approved by the investigators, local regulatory authorities, and the Ethics Committee. Following approval, data can be transferred through a secure online platform after signing a data access agreement and a confidentiality agreement.

## Supplementary Materials

The following supporting information can be downloaded at https://www.mdpi.com/article/10.3390/ijms27209116/s1.

## Abbreviations

The following abbreviations are used in this manuscript:

BMI	Body mass index
CAD	Coronary artery disease
CCC	Coronary collateral circulation
COPD	Chronic obstructive pulmonary disease
CTO	Chronic total occlusion
CVD	Cardiovascular diseases
DE	Differentially expressed
HDL	High-density lipoprotein
LDL	Low-density lipoprotein
Me	Median
miRNA	microRNA
NGS	Next-generation sequencing
NMRC	National Medical Research Center
*p*adj	*p*-values adjusted for multiple comparisons
PCA	Principal component analysis
PCR	Polymerase chain reaction
Q	Quartile
qPCR	Quantitative real-time polymerase chain reaction
UMI	Unique molecular identifiers

## References

[1] Brilakis E.S., Mashayekhi K., Tsuchikane E., Abi Rafeh N., Alaswad K., Araya M., Avran A., Azzalini L., Babunashvili A.M., Bayani B. (2019). Guiding Principles for Chronic Total Occlusion Percutaneous Coronary Intervention. Circulation.

[2] Råmunddal T., Hoebers L.P., Henriques J.P.S., Dworeck C., Angerås O., Odenstedt J., Ioanes D., Olivecrona G., Harnek J., Jensen U. (2014). Chronic Total Occlusions in Sweden—A Report from the Swedish Coronary Angiography and Angioplasty Registry (SCAAR). PLoS ONE.

[3] Fefer P., Knudtson M.L., Cheema A.N., Galbraith P.D., Osherov A.B., Yalonetsky S., Gannot S., Samuel M., Weisbrod M., Bierstone D. (2012). Current Perspectives on Coronary Chronic Total Occlusions: The Canadian Multicenter Chronic Total Occlusions Registry. J. Am. Coll. Cardiol..

[4] Christofferson R.D., Lehmann K.G., Martin G.V., Every N., Caldwell J.H., Kapadia S.R. (2005). Effect of Chronic Total Coronary Occlusion on Treatment Strategy. Am. J. Cardiol..

[5] Zimarino M., D’Andreamatteo M., Waksman R., Epstein S.E., De Caterina R. (2014). The Dynamics of the Coronary Collateral Circulation. Nat. Rev. Cardiol..

[6] Hakimzadeh N., Nossent A.Y., van der Laan A.M., Schirmer S.H., de Ronde M.W.J., Pinto-Sietsma S.-J., van Royen N., Quax P.H.A., Hoefer I.E., Piek J.J. (2015). Circulating MicroRNAs Characterizing Patients with Insufficient Coronary Collateral Artery Function. PLoS ONE.

[7] Hakimzadeh N., Elias J., Wijntjens G.W.M., Theunissen R., van Weert A., Smulders M.W., van den Akker N., Moerland P.D., Verberne H.J., Hoebers L.P. (2017). Monocytic microRNA Profile Associated with Coronary Collateral Artery Function in Chronic Total Occlusion Patients. Sci. Rep..

[8] Gao W., Zhang J., Wu R., Yuan J., Ge J. (2022). Integrated Analysis of Angiogenesis Related lncRNA-miRNA-mRNA in Patients With Coronary Chronic Total Occlusion Disease. Front. Genet..

[9] Vural M.G., Temel H.Y., Turunc E., Akdemir R., Tatli E., Agac M.T. (2024). Transcoronary Gradients of Mechanosensitive MicroRNAs as Predictors of Collateral Development in Chronic Total Occlusion. Medicina.

[10] Rentrop K.P., Cohen M., Blanke H., Phillips R.A. (1985). Changes in Collateral Channel Filling Immediately after Controlled Coronary Artery Occlusion by an Angioplasty Balloon in Human Subjects. J. Am. Coll. Cardiol..

[11] Fei Y., Hou J., Xuan W., Zhang C., Meng X. (2018). The Relationship of Plasma miR-503 and Coronary Collateral Circulation in Patients with Coronary Artery Disease. Life Sci..

[12] Ma X., Mei S., He Y., Wuyun Q., Zhou L., Cai Z., Luo Q., Wen Y., Yan J. (2025). Unraveling the Association and Regulatory Role of miR-146b-5p in Coronary Artery Disease. BMC Cardiovasc. Disord..

[13] Wang J., Yan Y., Song D., Liu B. (2016). Reduced Plasma miR-146a Is a Predictor of Poor Coronary Collateral Circulation in Patients with Coronary Artery Disease. BioMed Res. Int..

[14] Zhu L.-P., Zhou J.-P., Zhang J.-X., Wang J.-Y., Wang Z.-Y., Pan M., Li L.-F., Li C.-C., Wang K.-K., Bai Y.-P. (2017). MiR-15b-5p Regulates Collateral Artery Formation by Targeting AKT3 (Protein Kinase B-3). Arterioscler. Thromb. Vasc. Biol..

[15] Kiseleva A.V., Sotnikova E.A., Kutsenko V.A., Zharikova A.A., Ershova A.I., Meshkov A.N., Drapkina O.M. (2024). Circulating microRNAs and Collateral Circulation in Coronary Chronic Total Occlusion. Cardiovasc. Ther. Prev..

[16] Sotnikova E.A., Kiseleva A.V., Meshkov A.N. (2024). Preanalytical Factors Affecting the Plasma and Serum Levels of Circulating microRNAs. Cardiovasc. Ther. Prev..

[17] Mussbacher M., Krammer T.L., Heber S., Schrottmaier W.C., Zeibig S., Holthoff H.-P., Pereyra D., Starlinger P., Hackl M., Assinger A. (2020). Impact of Anticoagulation and Sample Processing on the Quantification of Human Blood-Derived microRNA Signatures. Cells.

[18] Zhelankin A.V., Iulmetova L.N., Sharova E.I. (2022). The Impact of the Anticoagulant Type in Blood Collection Tubes on Circulating Extracellular Plasma MicroRNA Profiles Revealed by Small RNA Sequencing. Int. J. Mol. Sci..

[19] Suzuki K., Yamaguchi T., Kohda M., Tanaka M., Takemura H., Wakita M., Tabe Y., Kato S., Nasu M., Hashimoto T. (2022). Establishment of Preanalytical Conditions for microRNA Profile Analysis of Clinical Plasma Samples. PLoS ONE.

[20] Khoo V., Shen L., Zhao L., Khoo V., Loo G., Richards A.M., Yeo T.-C., Lee C.-H. (2014). Determination of the Severity of Underlying Lesions in Acute Myocardial Infarction on the Basis of Collateral Vessel Development. Coron. Artery Dis..

[21] Kozomara A., Birgaoanu M., Griffiths-Jones S. (2019). miRBase: From microRNA Sequences to Function. Nucleic Acids Res..

[22] Kiseleva A.V., Vasilyev D.K., Soplenkova A.G., Shukurov F.B., Sotnikova E.A., Feshchenko D.A., Kutsenko V.A., Arablinsky N.A., Skirko O.P., Zharikova A.A. (2024). Association of Plasma microRNA Levels with Different Collateral Circulation Degree in Chronic Total Occlusion Patients with Coronary Artery Disease: A Pilot Study. Cardiovasc. Ther. Prev..

[23] Aleksander S.A., Balhoff J., Carbon S., Cherry J.M., Drabkin H.J., Ebert D., Feuermann M., Gaudet P., Harris N.L., Gene Ontology Consortium (2023). The Gene Ontology Knowledgebase in 2023. Genetics.

[24] Kanehisa M., Furumichi M., Sato Y., Kawashima M., Ishiguro-Watanabe M. (2023). KEGG for Taxonomy-Based Analysis of Pathways and Genomes. Nucleic Acids Res..

[25] McGeary S.E., Lin K.S., Shi C.Y., Pham T.M., Bisaria N., Kelley G.M., Bartel D.P. (2019). The Biochemical Basis of microRNA Targeting Efficacy. Science.

[26] Kiseleva A.V., Sotnikova E.A., Zharikova A.A., Kutsenko V.A., Borisova A.L., Shalnova S.A., Ershova A.I., Meshkov A.N., Drapkina O.M. (2025). Optimization of a Library Preparation Protocol for Sequencing Circulating Plasma and Serum microRNAs. Cardiovasc. Ther. Prev..

[27] Zhang L., Zhou M., Qin G., Weintraub N.L., Tang Y. (2014). MiR-92a Regulates Viability and Angiogenesis of Endothelial Cells under Oxidative Stress. Biochem. Biophys. Res. Commun..

[28] Zhelankin A.V., Vasiliev S.V., Stonogina D.A., Babalyan K.A., Sharova E.I., Doludin Y.V., Shchekochikhin D.Y., Generozov E.V., Akselrod A.S. (2020). Elevated Plasma Levels of Circulating Extracellular miR-320a-3p in Patients with Paroxysmal Atrial Fibrillation. Int. J. Mol. Sci..

[29] Sotnikova E.A., Kiseleva A.V., Meshkov A.N. (2024). Effect of Plasma and Serum Storage Conditions on Circulating microRNA Levels. Cardiovasc. Ther. Prev..

[30] Mikhailina V.I., Meshkov A.N., Kiseleva A.N., Ershova A.I., Drapkina O.M. (2024). MicroRNA as Biomarkers of Coronary Artery Disease in Real-World Practice. Cardiovasc. Ther. Prev..

[31] Page K., Guttery D.S., Zahra N., Primrose L., Elshaw S.R., Pringle J.H., Blighe K., Marchese S.D., Hills A., Woodley L. (2013). Influence of Plasma Processing on Recovery and Analysis of Circulating Nucleic Acids. PLoS ONE.

[32] Balzano F., Deiana M., Dei Giudici S., Oggiano A., Baralla A., Pasella S., Mannu A., Pescatori M., Porcu B., Fanciulli G. (2015). miRNA Stability in Frozen Plasma Samples. Molecules.

[33] Kiseleva A.V., Sotnikova E.A., Zharikova A.A., Kutsenko V.A., Vasiliev D.K., Pokrovskaya M.S., Ershova A.I., Meshkov A.N., Drapkina O.M. (2025). Effect of Long-Term Plasma Storage on the Profile of Circulating microRNAs. Cardiovasc. Ther. Prev..

[34] Kegler U., Ropek N., Hofner M., Schönthaler S., Vierlinger K., Nöhammer C. (2025). Optimizing Small RNA Sequencing for Salivary Biomarker Identification: A Comparative Study of Library Preparation Protocols. Int. J. Mol. Sci..

[35] Androvic P., Benesova S., Rohlova E., Kubista M., Valihrach L. (2022). Small RNA-Sequencing for Analysis of Circulating miRNAs: Benchmark Study. J. Mol. Diagn..

[36] (2006). Good Clinical Practice.

[37] Zharikova A.A., Vyatkin Y.V., Kiseleva A.V., Meshkov A.N. (2024). Bioinformatics Approach to Processing Data from High-Throughput Sequencing of Small RNA Molecules. Cardiovasc. Ther. Prev..

[38] Smith T., Heger A., Sudbery I. (2017). UMI-Tools: Modeling Sequencing Errors in Unique Molecular Identifiers to Improve Quantification Accuracy. Genome Res..

[39] Martin M. (2011). Cutadapt Removes Adapter Sequences from High-Throughput Sequencing Reads. EMBnet J..

[40] Dobin A., Davis C.A., Schlesinger F., Drenkow J., Zaleski C., Jha S., Batut P., Chaisson M., Gingeras T.R. (2013). STAR: Ultrafast Universal RNA-Seq Aligner. Bioinformatics.

[41] Danecek P., Bonfield J.K., Liddle J., Marshall J., Ohan V., Pollard M.O., Whitwham A., Keane T., McCarthy S.A., Davies R.M. (2021). Twelve Years of SAMtools and BCFtools. Gigascience.

[42] Liao Y., Smyth G.K., Shi W. (2014). featureCounts: An Efficient General Purpose Program for Assigning Sequence Reads to Genomic Features. Bioinformatics.

[43] Wickham H. (2009). Ggplot2: Elegant Graphics for Data Analysis.

[44] Love M.I., Huber W., Anders S. (2014). Moderated Estimation of Fold Change and Dispersion for RNA-Seq Data with DESeq2. Genome Biol..

