# Supplementary material for: Association of Circulating microRNAs in Plasma with Coronary Collateral Circulation in Patients with Chronic Total Occlusion
Source: Int J Mol Sci. 2026 Oct 14;27(20):9116. doi: 10.3390/ijms27209116 (PMC13705095; doi:10.3390/ijms27209116)
Supplement: Supplementary file 1 [file ijms-27-09116-s001.zip › Supplemetary figures.pdf]

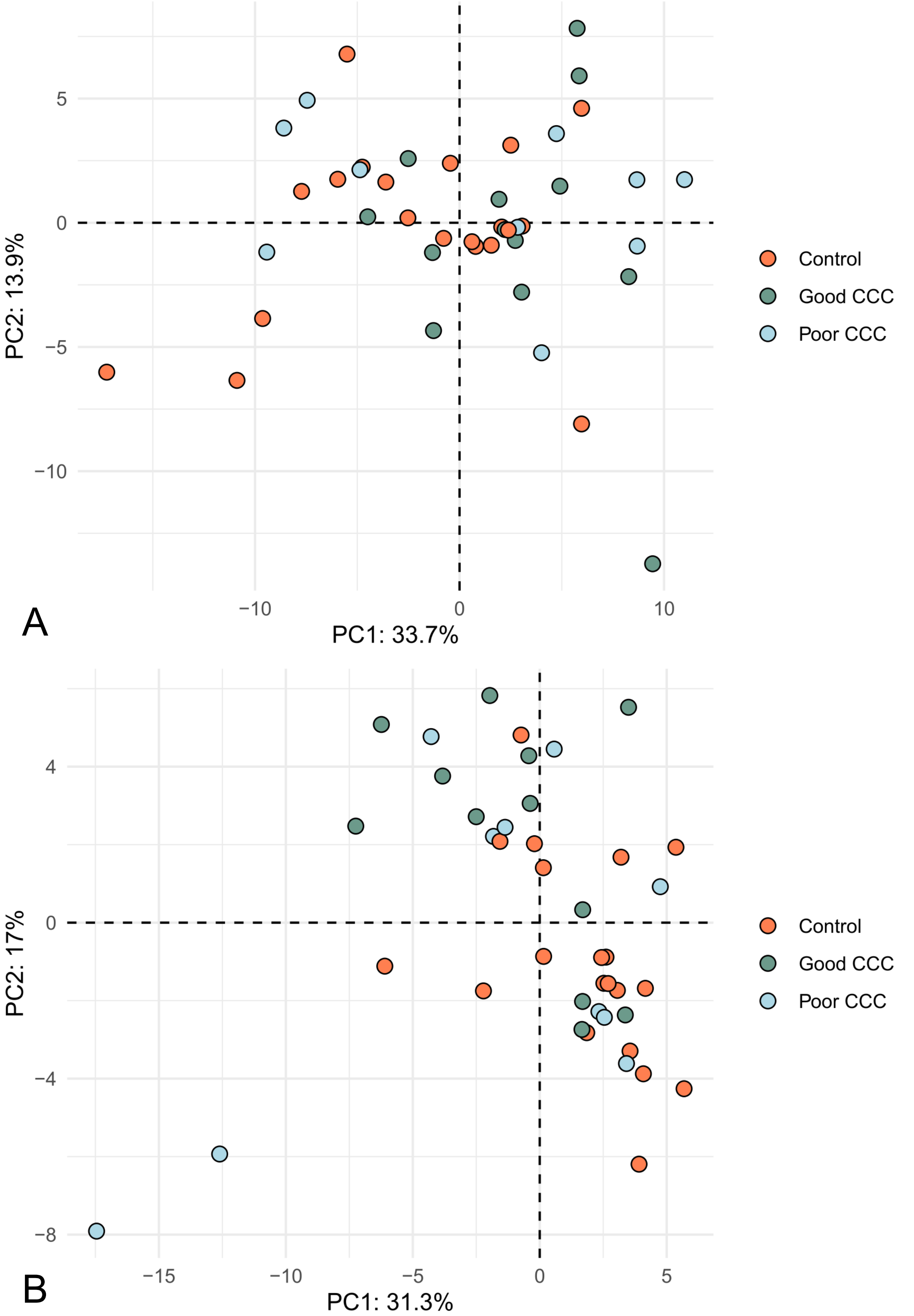

Supplementary Figure S1. Principal component analysis of the comparison groups. (**A**) EDTA plasma samples; (**B**) citrate plasma samples. The good CCC group is colored green; the poor CCC group —blue; the control group —orange. CCC—coronary collateral circulation; CTO—chronic total occlusion of a coronary artery.

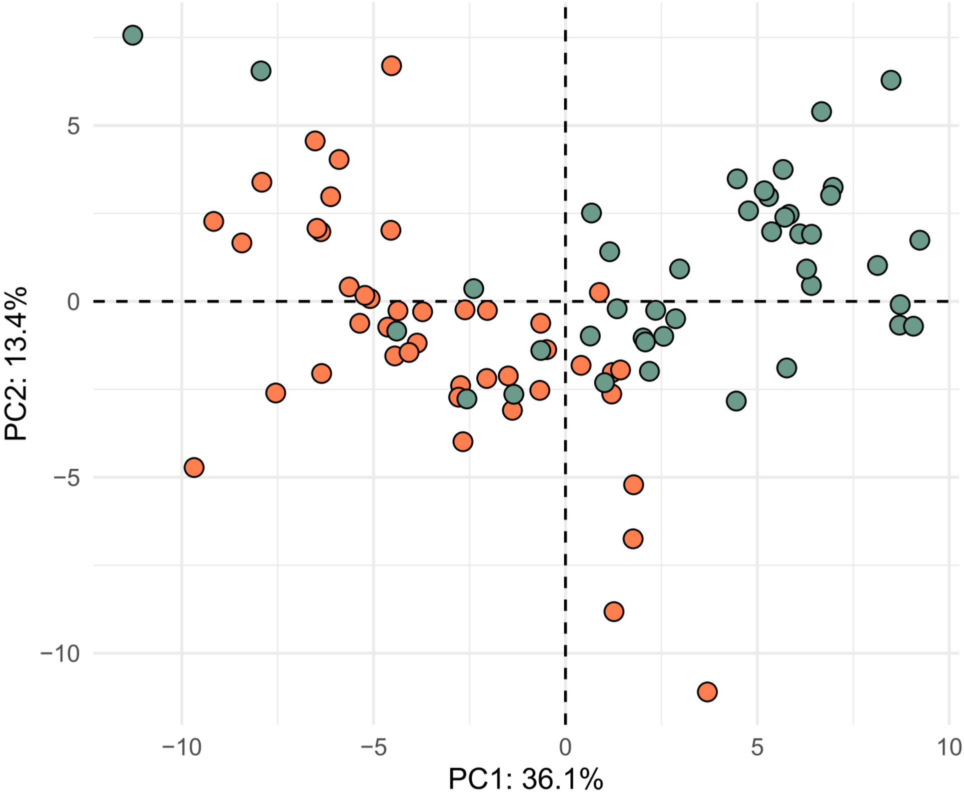

Supplementary Figure S2. Principal component analysis of the comparison group. EDTA plasma samples are colored orange; citrate plasma samples — green.
